# Clinical course of 12 patients on a Covid-19 dementia isolation ward

**DOI:** 10.1192/bjb.2020.109

**Published:** 2020-12

**Authors:** Richard Kerslake, Scott Cherry, Jessica Buckle, Richard Harris, Richard Caplan

**Affiliations:** Psychiatry Higher Specialist Trainee, RCPsych; email: richard.kerslake@nhs.net; Consultant Psychiatrist, Sussex Partnership NHS Foundation Trust; Psychiatry Core Trainee, @sussexpartnership.nhs.uk; Psychiatry Core Trainee, @sussexpartnership.nhs.uk; Medical Student, Brighton & Sussex Medical School

Older adults have been particularly vulnerable to outbreaks of severe acute respiratory syndrome coronavirus 2 (Covid-19), and mortality rates increase with age.^1,2^ Physiological comorbidities and nutritional status are contributory factors, but there is limited understanding of the influence of mental illness, particularly for patients with dementia.^3–5^ Furthermore, cognitive impairment may also increase the risks of contracting Covid-19.^6^

We have summarised our experiences from a psychiatric in-patient unit to describe the clinical outcomes of 12 patients admitted to a dementia specialist ward, which later became closed to admissions after nine of the patients were isolated with confirmed or suspected Covid-19. The following patients, all over the age of 74, were selected to illustrate the typical clinical course that was observed in the most unwell patients. To ensure anonymity, patient initials, ages, and gender identifiers have been removed.

In Case 1, the patient had diagnoses of mixed Alzheimer's and vascular dementia, type 2 diabetes mellitus, hypertension and previous traumatic subdural haemorrhage. Isolation was commenced after a new cough, low oxygen saturations and vomiting were observed. The patient was then transferred to the acute hospital and treated with empirical antibiotics. Despite this, the patient refused all oral food and fluids, and a decrease of 24% of pre-Covid total body mass was recorded. The patient later developed hypernatraemia and an ischaemic left leg and was subsequently discharged to a palliative care hospice on an end-of-life care pathway.

In Case 2, the patient with a diagnosis of mixed Alzheimer's and vascular dementia and no relevant past medical history developed a cough and oxygen saturations of 88% on air. The patient was transferred to the acute hospital, diagnosed with lobar pneumonia, started on antibiotics and returned to the ward 5 days later. Throughout the period of isolation, food and fluid intake was observed as being minimal. Twelve days after isolation, a serum sodium level of 147 mmol/L was recorded, with a decrease of 12.7% of pre-Covid body mass. Features of pneumonia and dehydration persisted, with the serum sodium level climbing to 152 mmol/L. Following three further episodes of care at the acute hospital, the patient was treated on an end-of-life care pathway and died 30 days after isolation. Of note, 4 days before isolation was commenced, this patient was medically reviewed for a cough which was thought not to be due to Covid-19. Causes of death were certified as (1a) pneumonia, (1b) Covid-19 infection and (2) end-stage dementia.

In Case 3, the patient had a previous acquired brain injury and mixed Alzheimer's and vascular dementia, with past medical history including epilepsy and chronic obstructive pulmonary disease. Isolation nursing commenced after a new cough was noted, 5 days after a possible cough was interpreted as ‘throat clearing’. Eleven days after isolation, the patient was initiated on antibiotics at the acute hospital after a significant drop in oxygen saturations was recorded. Serum sodium levels were sampled at 151 mmol/L, corresponding with a limited fluid intake. A decrease of 23% of pre-Covid body mass was recorded. Thirteen days after isolation, the patient developed hyperactive delirium with clinical signs of dehydration, serum sodium of 156 mmol/l and urea of 15.1 mmol/L. The patient was subsequently transferred to the acute hospital and placed on an end-of-life care pathway.

In Case 4, the patient had diagnoses of Alzheimer's dementia and treatment-resistant depression, having been admitted from the acute hospital. Isolation was commenced, followed by transfer back to the acute hospital, after oxygen saturations dropped to 90%. After correction of hypokalaemia and commencing antibiotics for pneumonia, the patient returned to the ward. Six days after the initial Covid-19 symptoms, the patient was readmitted to the acute hospital for management of reduced oxygen saturation and refeeding syndrome. A further 21 days later, the patient was placed on an end-of-life care pathway. The cause of death was recorded as (1a) end-stage dementia in Alzheimer's disease and Covid-19 infection and (2) urinary tract infection (treated).

All isolated patients with reduced mobility were prescribed venous thromboembolism prophylaxis. All isolated patients with a cough or fever were prescribed prophylactic antibiotics in case of a secondary bacterial pneumonia. All confirmed or suspected Covid patients were prescribed additional nutritional supplements. These plans were implemented with advice from acute hospital and dietician colleagues.

Of the remaining eight patients on the ward, one was asymptomatic throughout the 14 days of isolation despite testing positive for the virus, and four further patients tested positive, requiring various levels of medical input before being safely discharged. A further three patients did not record a fever, nor develop a cough, and did not test positive on throat or nasal swabs and were nursed separately.

In summary, of the nine patients who were confirmed or suspected of having contracted Covid-19, four subsequently received end-of-life care, eight required admission to an acute hospital for medical input, and all nine patients survived the period of isolation, which lasted at least 14 days.

All confirmed or suspected Covid patients were prescribed additional nutritional supplements under the guidance of a dietician. Adherence to these was variable but generally poor. Despite this, four patients experienced a weight loss of between 13% to 24% during their period of isolation. This weight loss was observed in the patients with the poorest outcomes.

Six patients also experienced clinical levels of dehydration, identified initially by raised serum sodium levels, despite best attempts to encourage oral fluids while being nursed in isolation. These patients also required the highest levels of medical input.

In our opinion, patients with dementia who contract Covid-19 are likely to require high levels of medical oversight to monitor their physical health, experienced nursing care to encourage adequate fluid and food intake, and input from a dietician to ensure nutrition is optimised.

The clinical outcomes of patients with dementia who contract Covid-19 are variable and difficult to predict. It can be expected that patients will require episodes of care in an acute hospital for physical consequences of Covid-19 that cannot be addressed on a psychiatric ward. Physical deterioration and death do not tend to occur during the 14 days of isolation and are usually due to secondary consequences of Covid-19, of which weight loss, dehydration and rising serum sodium are worrying clinical signs.

Self-reporting of Covid-19 symptoms by patients with dementia can be unreliable and can delay isolation nursing, putting other patients at risk.

Management of symptomatic patients who have refused confirmatory swabs must be carefully considered, balancing the risks and benefits of treatment in the Covid-19 cohort area or treatment among well patients.

Early discussions related to ceilings of care, Do Not Attempt Cardio Pulmonary Resuscitation orders and the application of end-of-life care pathways were recognised as essential to ensuring that the patient's prior wishes and best interests were considered.

We sincerely hope that the details we have included here will be of interest and use to readers. We will happily respond to any questions that may arise.
